# Aneurism of the Aorta

**Published:** 1899-12-16

**Authors:** 


### ANEURISM OF THE AORTA.

Speaking at the London Polyclinic upon a case of aneurism of the arch of the aorta, Sir William Broadbent pointed out the very different effects produced by that disease according as it affected the earlier or the later portion of the arch of the aorta. The ascending aorta and the first part of the arch move freely up and down with each beat of the heart, and this mobility is provided for by the anatomical arrangements of the part. In consequence of such provision an aneurism of this part of the great vessel may attain considerable size, and betray its presence by conspicuous physical signs without pressing upon any important structure. Aneurism of the transverse or descending division of the arch, on the other hand, very soon implicates nerves or veins, or the trachea, or the root of the left lung, giving rise to symptoms even while the physical signs are still obscure and difficult of recognition. Hence, while aneurism of the first part gives rise to the condition spoken of as "aneurism of physical signs," aneurism of the later part of the aortic arch gives rise to "aneurism of symptoms." Passing over the symptoms discovered in this case, we may consider what Sir William Broadbent has to say in regard to prognosis. An aneurism of the ascending arch may reach a very large size without pressing on any important organ, and, therefore, without giving rise to those secondary effects which produce suffering and shorten life, and as a matter of personal experience he said that the cases which had survived the longest had been very similar to the one now under discussion, which was of the class of "aneurism of physical signs." Another reason for a comparatively favourable prognosis in this case was that percussion showed the aneurism to be of some size, while the neck was comparatively narrow (there being good resonance in the second space close to the sternum), for, paradoxical as it might appear, there is a better chance of cure of a large aneurism than of a small one. Where the sac is small the communication with the artery is relatively large, and the blood current will sweep through the aneurism and effectually prevent the deposit of fibrin, while with the large aneurism, with a comparatively small neck, the blood contained in the aneurism is withdrawn from that which is rushing along the aorta and is comparatively stagnant, which affords the desired opportunity for the deposit of laminated clot.

